# Supplementary material for: Genomic signatures of recent convergent transitions to social life in spiders
Source: Nat Commun. 2022 Nov 22;13:6967. doi: 10.1038/s41467-022-34446-8 (PMC9681848; doi:10.1038/s41467-022-34446-8)
Supplement: Supplementary file 1 — Supplementary Information [file 41467_2022_34446_MOESM1_ESM.pdf]

Supplementary Information

Genomic signatures of recent convergent transitions to social life in spiders

Chao Tong, Leticia Avilés, Linda S. Rayor, Alexander S. Mikheyev, Timothy A. Linksvayer

Corresponding authors: Chao Tong, Timothy A. Linksvayer
Email: tongchao1990@gmail.com (C.T.), tlinksvayer@gmail.com (T.A.L.)

Supplementary Figures

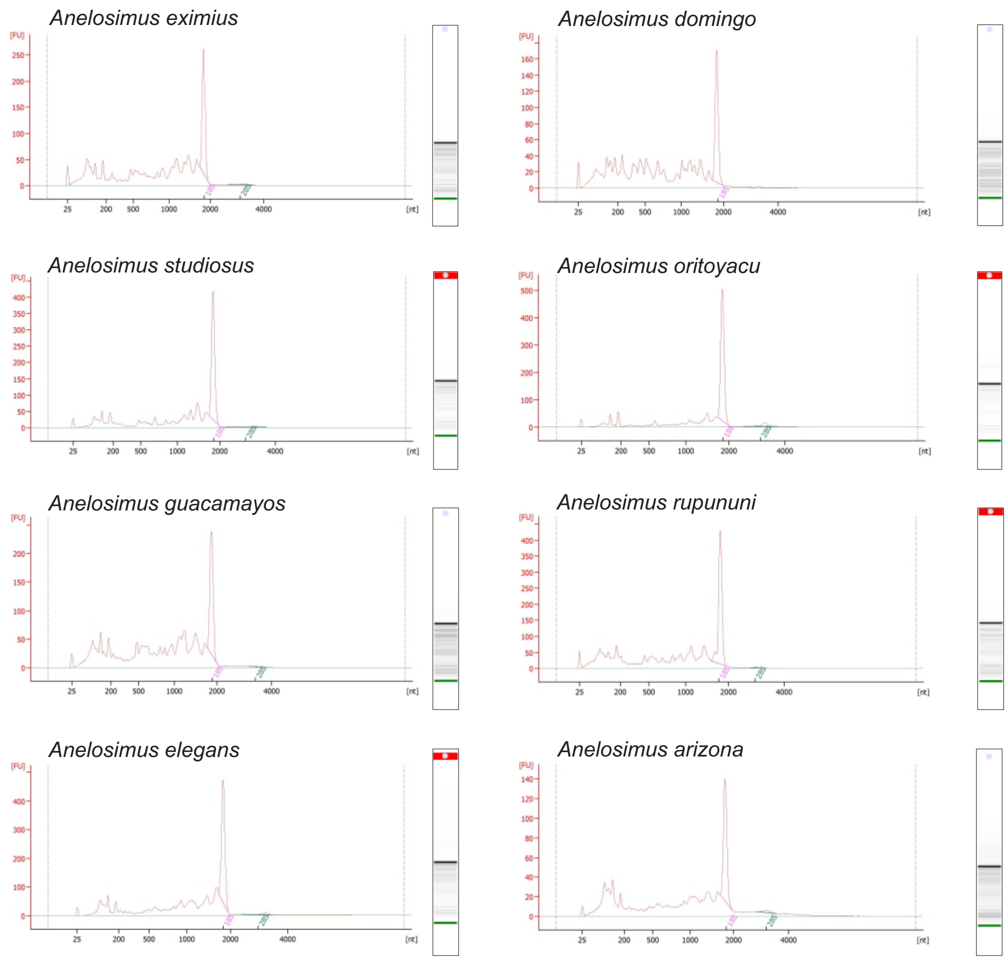

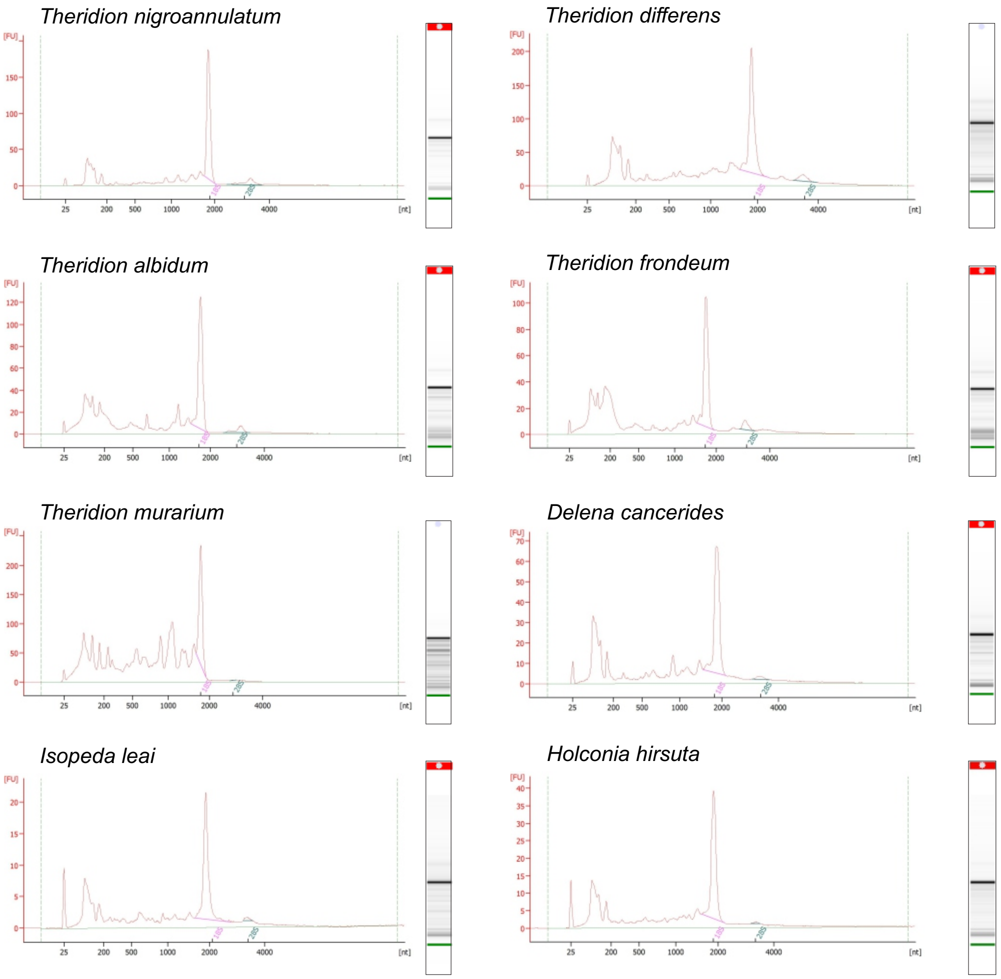

Supplementary Figure 1. The quality of 16 spider RNA extracted from ethanol preserved specimens. All RNA samples were assessed with Agilent Bioanalyzer 2100 by using Agilent RNA 6000 Pico Kit.

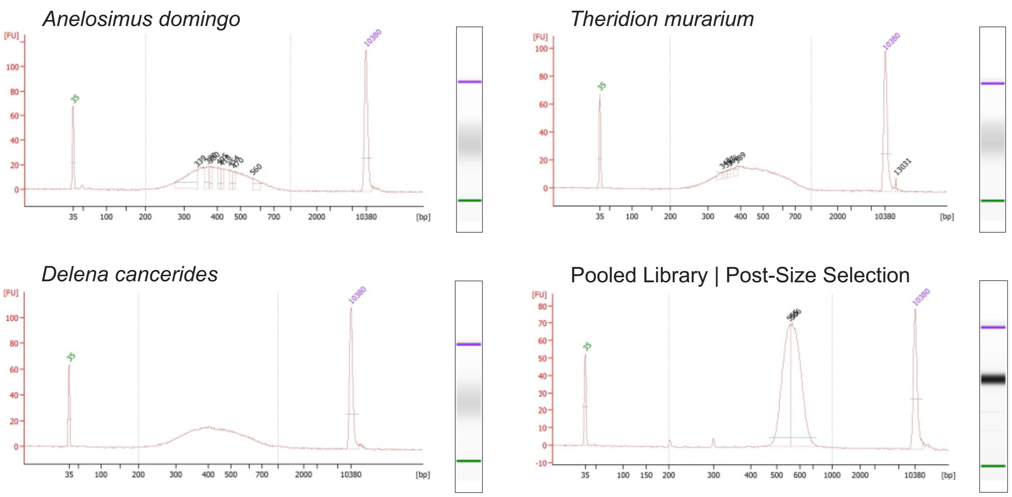

Supplementary Figure 2. The quality and size distribution of constructed RNA sequencing libraries. Three representative constructed RNA-seq libraries from three clades, including genera *Anelosimus* and *Theridion* (Theridiidae), and *Delena* (Sparassidae), and final pooled RNA-seq library after size selection were assessed with Agilent Bioanalyzer 2100 by using Agilent High Sensitivity DNA Kit.

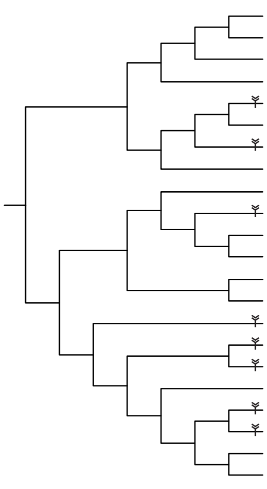

Positions	168	233	264	301	307	310	372	
Reference	S	E	P	A	P	H	K	
I. leai	●	●	●	●	●	●	●	solitary
Ho. hirsuta	●	●	●	●	●	●	●	solitary
D. cancerides	P	V	L	V	●	●	●	prolonged subsocial
He. sp. ATS12	●	●	●	●	●	●	●	solitary
S. mimosarum	P	●	●	●	●	●	●	social
S. africanus	●	●	●	●	●	●	●	subsocial
S. dumicola	●	V	L	V	H	Y	R	social
S. lineatus	●	●	●	●	●	●	●	subsocial
T. grallator	●	●	●	●	●	●	●	solitary
T. nigroannulatum	P	V	L	V	H	Y	R	social
T. albidum	●	●	●	●	●	●	●	solitary
T. frondeum	●	●	●	●	●	●	●	solitary
T. differens	●	●	●	●	●	●	●	solitary
T. murarium	●	●	S	●	●	●	●	solitary
A. rupununi	●	●	●	●	●	●	●	social
A. eximius	P	V	L	V	H	Y	R	social
A. domingo	●	●	●	●	●	●	●	social
A. studiosus	●	●	●	●	●	●	●	subsocial
A. guacamayos	●	●	●	●	●	●	●	social
A. oritoyacu	P	V	L	V	H	Y	R	social
A. arizona	P	V	L	V	H	Y	R	subsocial
A. elegans	●	●	●	●	●	●	●	subsocial

Supplementary Figure 3. Convergent amino acid substitution at sites in Bromodomain Containing 4 (Brd4) in social spider taxa. The arrow tails on the phylogenetic tree represent the independent origins of sociality in spiders. Orange-shaded rows correspond to social spider species. The gray-shaded row represents the only prolonged subsocial spider species. Numbered columns correspond to convergent substitution sites in social spider taxa. The dark dots represent the identical amino acid sites corresponding to reference sites above.

Supplementary Tables

Supplementary Table 1. Sample information and geographic locations.

Species name	Initial	Family	Primary social organization	Secondary social organization	Specimen/Date source	Materials	Preservation	Country	State	Locatity	Collector	Date
Anelosimus eximius	Aexi	Theridiidae	social	social	Newly collected	Whole body	95% ethanol	Ecuador	Napo	Lyarina Lodge	Leticia Avilés	2017
Anelosimus domingo	Adom	Theridiidae	social	social	Newly collected	Whole body	95% ethanol	Ecuador	Napo	Jatun Sacha Biological Reserve	Leticia Avilés	2017
Anelosimus studiosus	Astu	Theridiidae	subsocial	nonsocial	Newly collected	Whole body	95% ethanol	USA	Tennessee	Tennessee Valley Authority	Leticia Avilés	2017
Anelosimus oritoyacu	Aori	Theridiidae	social	social	Newly collected	Whole body	95% ethanol	Ecuador	Napo	Baeza + 2.7km	Leticia Avilés	5/23/2015
Anelosimus guacamayos	Agua	Theridiidae	social	social	Newly collected	Whole body	95% ethanol	Ecuador	Napo	Cocodrillos, 6.7km S La Virgen	Leticia Avilés	7/20/2018
Anelosimus rupununi	Arup	Theridiidae	social	social	Newly collected	Whole body	95% ethanol	Ecuador	Pastaza	28km S of Tena	Leticia Avilés	6/8/2009
Anelosimus elegans	Aele	Theridiidae	subsocial	nonsocial	Newly collected	Whole body	95% ethanol	Ecuador	Napo	Via a Loreto, km 17.3	Leticia Avilés	2017
Anelosimus arizona	Aari	Theridiidae	subsocial	nonsocial	Newly collected	Whole body	95% ethanol	USA	Arizona	Santa Cruz County	Leticia Avilés	9/22/2018
Theridion nigroannulatum	Tnig	Theridiidae	social	social	Newly collected	Whole body	70% ethanol	Ecuador	Napo	Antisana Ecological Reserve	Leticia Avilés	2017
Theridion differens	Tdif	Theridiidae	solitary	nonsocial	Newly collected	Whole body	70% ethanol	USA	Indiana	McVey Memorial Forest, Randolph County	Marc Milne	5/22/2014
Theridion albidum	Talb	Theridiidae	solitary	nonsocial	Newly collected	Whole body	70% ethanol	USA	Indiana	Yellowwood State Forest, Brown County	Marc Milne	8/16/2017
Theridion frondeum	Tfro	Theridiidae	solitary	nonsocial	Newly collected	Whole body	70% ethanol	USA	Indiana	Mitchell, Lawrence County	Marc Milne	8/24/2017
Theridion murarium	Tmur	Theridiidae	solitary	nonsocial	Newly collected	Whole body	70% ethanol	USA	Indiana	Indiana Dunes National Park	Marc Milne	6/13/2018
Theridion grallator	Tgra	Theridiidae	solitary	nonsocial	NCBI downloaded	N/A	N/A	N/A	N/A	N/A	N/A	N/A
Delena cancerides	Dcan	Sparassidae	prolonged subsocial	nonsocial	Newly collected	Whole body	95% ethanol	Australia	Australian Capital Territory	Canberra	Linda Rayor	2017

Species name	Initial	Family	Primary social organization	Secondary social organization	Specimen/Date source	Materials	Preservation	Country	State	Locatity	Collector	Date
Isopeda leai	Ilea	Sparassidae	solitary	nonsocial	Newly collected	Whole body	95% ethanol	Australia	South Australia	Adelaide	Linda Rayor	2017
Holconia hirsuta	Hhis	Sparassidae	solitary	nonsocial	Newly collected	Whole body	95% ethanol	Australia	Queensland	Mareeba	Linda Rayor	2017
Heteropoda sp. ATS12	Hesp	Sparassidae	solitary	nonsocial	NCBI downloaded	N/A	N/A	N/A	N/A	N/A	N/A	N/A
Stegodyphus mimosarum	Smim	Eresidae	social	social	NCBI downloaded	N/A	N/A	N/A	N/A	N/A	N/A	N/A
Stegodyphus dumicola	Sdum	Eresidae	social	social	NCBI downloaded	N/A	N/A	N/A	N/A	N/A	N/A	N/A
Stegodyphus africanus	Safr	Eresidae	subsocial	nonsocial	NCBI downloaded	N/A	N/A	N/A	N/A	N/A	N/A	N/A
Stegodyphus lineatus	Slin	Eresidae	subsocial	nonsocial	NCBI downloaded	N/A	N/A	N/A	N/A	N/A	N/A	N/A

Supplementary Table 2. Information for additional genome or transcriptome of closely related spider species.

Species name	Initial	Family	Primary social organization	Secondary social organization	Data source	Data type	NCBI Accession Number
Anelosimus eximius	Aexi	Theridiidae	social	social	Newly Sequenced	Transcriptome	PRJNA685164
Anelosimus domingo	Adom	Theridiidae	social	social	Newly Sequenced	Transcriptome	PRJNA685165
Anelosimus studiosus	Astu	Theridiidae	subsocial	nonsocial	NCBI downloaded	Whole Genome	GCA_008297655.1
					Newly Sequenced	Transcriptome	PRJNA685165
Anelosimus oritoyacu	Aori	Theridiidae	social	social	Newly Sequenced	Transcriptome	PRJNA685166
Anelosimus guacamayos	Agua	Theridiidae	social	social	Newly Sequenced	Transcriptome	PRJNA685167
Anelosimus rupununi	Arup	Theridiidae	social	social	Newly Sequenced	Transcriptome	PRJNA685168
Anelosimus elegans	Aele	Theridiidae	subsocial	nonsocial	Newly Sequenced	Transcriptome	PRJNA685169
Anelosimus arizona	Aari	Theridiidae	subsocial	nonsocial	Newly Sequenced	Transcriptome	PRJNA685170
Theridion nigroannulatum	Tnig	Theridiidae	social	social	Newly Sequenced	Transcriptome	PRJNA685171
Theridion differens	Tdif	Theridiidae	solitary	nonsocial	Newly Sequenced	Transcriptome	PRJNA685172
Theridion albidum	Talb	Theridiidae	solitary	nonsocial	Newly Sequenced	Transcriptome	PRJNA685173
Theridion frondeum	Tfro	Theridiidae	solitary	nonsocial	Newly Sequenced	Transcriptome	PRJNA685174
Theridion murarium	Tmur	Theridiidae	solitary	nonsocial	Newly Sequenced	Transcriptome	PRJNA685175
Theridion grallator	Tgra	Theridiidae	solitary	nonsocial	NCBI downloaded	Transcriptome	SRR960715<br>SRR960716<br>SRR960718<br>SRR960719<br>SRR960611<br>SRR960612<br>SRR960614<br>SRR960615<br>SRR960616
Delena cancerides	Dcan	Sparassidae	prolonged subsocial	nonsocial	Newly Sequenced	Transcriptome	PRJNA685175

Species name	Initial	Family	Primary social organization	Secondary social organization	Data source	Data type	NCBI Accession Number
Isopeda leai	Ilea	Sparassidae	solitary	nonsocial	Newly Sequenced	Transcriptome	PRJNA685176
Holconia hirsuta	Hhis	Sparassidae	solitary	nonsocial	Newly Sequenced	Transcriptome	PRJNA685177
Heteropoda sp. ATS12	Hesp	Sparassidae	solitary	nonsocial	NCBI downloaded	Transcriptome	SRR6425926
Stegodyphus mimosarum	Smim	Eresidae	social	social	NCBI downloaded	Whole Genome	GCA_000611955.2
Stegodyphus dumicola	Sdum	Eresidae	social	social	NCBI downloaded	Whole Genome	GCF_010614865.1
Stegodyphus africanus	Safr	Eresidae	subsocial	nonsocial	NCBI downloaded	Transcriptome	SRR7062696
Stegodyphus lineatus	Slin	Eresidae	subsocial	nonsocial	NCBI downloaded	Transcriptome	SRR7062695
Acanthoscurria geniculata	outgroup	Theraphosidae	solitary	nonsocial	NCBI downloaded	Whole Genome	GCA_000661875.1

Supplementary Table 3. Statistics of assembly quality, protein coding genes, orthologous genes in spiders.

Species name	Initial	Data source	Data type	Number of scaffolds	Scaffold N50	BUSCO (arachnida_odb10, n = 2934)	Percentage of identified orthologs (BUSCO)	Number of protein coding genes	Number of orthologous groups (OMA + OrthoDB)
Anelosimus eximius	Aexi	Newly Sequenced	Transcriptome	N/A	N/A	Completed:83.6%, Fragmented:6.2%, Missing:10.2%	89.80%	53,362	6926
Anelosimus domingo	Adom	Newly Sequenced	Transcriptome	N/A	N/A	Completed:80.5%, Fragmented:5.8%, Missing:14.7%	86.30%	37,511	6629
Anelosimus studiosus	Astu	NCBI downloaded	Whole Genome	956,665	4,793	Completed:33.9%, Fragmented:10.7%, Missing:55.4%	NA	N/A	N/A
		New Reassembly	Whole Genome	103,010	10,841	Completed:80.4%, Fragmented:8.2%, Missing:11.4%	88.60%	78,189	7092
Anelosimus oritoyacu	Aori	Newly Sequenced	Transcriptome	N/A	N/A	Completed:87.9%, Fragmented:5.4%, Missing:6.7%	93.30%	88,643	7087
Anelosimus guacamayos	Agua	Newly Sequenced	Transcriptome	N/A	N/A	Completed:89.0%, Fragmented:4.0%, Missing:7.0%	93.30%	61,103	6879
Anelosimus rupununi	Arup	Newly Sequenced	Transcriptome	N/A	N/A	Completed:93.7%, Fragmented:2.5%, Missing:3.8%	96.20%	60,623	7069
Anelosimus elegans	Aele	Newly Sequenced	Transcriptome	N/A	N/A	Completed:82.8%, Fragmented:7.5%, Missing:9.7%	90.30%	66,498	6999
Anelosimus arizona	Aari	Newly Sequenced	Transcriptome	N/A	N/A	Completed:87.1%, Fragmented:6.2%, Missing:6.7%	93.30%	96,176	7,117
Theridion nigroannulatum	Tnig	Newly Sequenced	Transcriptome	N/A	N/A	Completed:80.1%, Fragmented:8.3%, Missing:11.6%	88.40%	49,465	6509
Theridion differens	Tdif	Newly Sequenced	Transcriptome	N/A	N/A	Completed:83.4%, Fragmented:7.7%, Missing:8.9%	91.10%	56,373	7214
Theridion albidum	Talb	Newly Sequenced	Transcriptome	N/A	N/A	Completed:80.2%, Fragmented:7.2%, Missing:12.6%	87.40%	83,685	6820
Theridion frondeum	Tfro	Newly Sequenced	Transcriptome	N/A	N/A	Completed:80.7%, Fragmented:6.3%, Missing:13.0%	87.00%	38,203	6547
Theridion murarium	Tmur	Newly Sequenced	Transcriptome	N/A	N/A	Completed:90.0%, Fragmented:4.2%, Missing:5.8%	94.20%	67,469	7222

Species name	Initial	Data source	Data type	Number of scaffolds	Scaffold N50	BUSCO (arachnida_odb10, n = 2934)	Percentage of identified orthologs (BUSCO)	Number of protein coding genes	Number of orthologous groups (OMA + OrthoDB)
Theridion grallator	Tgra	NCBI downloaded	Transcriptome	N/A	N/A	Completed:83.7%, Fragmented:2.9%, Missing:13.4%	86.60%	26,386	6497
Delena cancerides	Dcan	Newly Sequenced	Transcriptome	N/A	N/A	Completed:80.4%, Fragmented:8.2%, Missing:11.4%	88.60%	23,889	6185
Isopeda leai	Ilea	Newly Sequenced	Transcriptome	N/A	N/A	Completed:80.3%, Fragmented:4.5%, Missing:15.2%	84.80%	26,862	6165
Holconia hirsuta	Hhis	Newly Sequenced	Transcriptome	N/A	N/A	Completed:81.4%, Fragmented:3.2%, Missing:15.4%	84.60%	40,767	6475
Heteropoda sp. ATS12	Hesp	NCBI downloaded	Transcriptome	N/A	N/A	Completed:92.7%, Fragmented:2.1% ,Missing:5.2%	94.80%	25,492	6866
Stegodyphus mimosarum	Smim	NCBI downloaded	Whole Genome	68,653	480,636	Completed:97.2%, Fragmented:1.8%, Missing:1.0%	99.00%	27,135	7590
Stegodyphus dumicola	Sdum	NCBI downloaded	Whole Genome	16,531	254,130	Completed:92.5%, Fragmented:3.3%, Missing:4.2%	95.80%	29,944	7049
Stegodyphus africanus	Safr	NCBI downloaded	Transcriptome	N/A	N/A	Completed:93.5%, Fragmented:2.1%, Missing:4.4%	95.60%	19,224	6808
Stegodyphus lineatus	Slin	NCBI downloaded	Transcriptome	N/A	N/A	Completed:96.7%, Fragmented:1.6%, Missing:1.7%	98.30%	21,573	7054

Supplementary Table 4. List of genes showing acceleration or deceleration in relative evolutionary rates in social branches.

ogg_id	Type	Rho	N	*p*-value	*q*-value	permulation *p*-value	Permulation *q*-value	Gene
OG80S32P	Accelerated	0.5709224	21	0.002249296	0.985353677	0.000100261	0.092678464	general transcription factor II-I repeat domain-containing protein 2-like
OG8BVVF9	Accelerated	0.53708616	13	0.02810804	0.985353677	0	0	Protein SYS1 homolog
OG8KPWXB	Accelerated	0.46785713	33	0.00140615	0.985353677	0	0	Protein krueppel
OG8C8BD2	Accelerated	0.42176249	25	0.013085535	0.985353677	0	0	GTPase-activating protein skywalker
OG8CG2VG	Accelerated	0.38103291	43	0.002791695	0.985353677	0.0002	0.1479	Protein vav
OG8D55JG	Accelerated	0.36494721	41	0.005221959	0.985353677	0	0	Low-density lipoprotein receptor-related protein 6
OG8XSP9P	Accelerated	0.32974002	43	0.009672625	0.985353677	0	0	NAD(P) transhydrogenase, mitochondrial
OG81VNPH	Decelerated	-0.3639064	39	0.006670276	0.985353677	0.0001	0.092678464	Iron-sulfur cluster assembly enzyme ISCU, mitochondrial
OG8SBHJ6	Decelerated	-0.3700416	43	0.003689629	0.985353677	0	0	Phosphate carrier protein, mitochondrial
OG8V19MH	Decelerated	-0.41976	37	0.002336603	0.985353677	0.0002	0.1479	Coiled-coil domain-containing protein 177

Supplementary Table 5. List of Gene Ontology (GO) terms under convergent acceleration in social branches.

go_term_id	stat	*p*-value	*q*-value	permulation *p*-value	permulation *q*-value	go_term
GO:0030512	0.253406867	0.003637358	0.039586422	0	0	negative regulation of transforming growth factor beta receptor signaling pathway
GO:1903845	0.253406867	0.003637358	0.039586422	0	0	negative regulation of cellular response to transforming growth factor beta stimulus
GO:0090288	0.179228049	0.000698145	0.012035662	0	0	negative regulation of cellular response to growth factor stimulus
GO:0043068	0.065470569	0.02086067	0.129104164	0	0	positive regulation of programmed cell death
GO:0008219	0.05469556	0.018696564	0.120050081	0	0	cell death
GO:0090101	0.163598901	0.004707223	0.047296908	1.00E-04	0.037263636	negative regulation of transmembrane receptor protein serine/threonine kinase signaling pathway
GO:0045465	0.178482831	0.01680917	0.111749778	2.00E-04	0.054653333	R8 cell differentiation
GO:0090090	0.199476173	5.93E-05	0.001531198	3.00E-04	0.068316667	negative regulation of canonical Wnt receptor signaling pathway
GO:0010942	0.055849929	0.03701149	0.188643656	3.00E-04	0.068316667	positive regulation of cell death
GO:0035017	0.203405935	0.000200868	0.004360643	4.00E-04	0.086294737	cuticle pattern formation
GO:0012502	0.245653096	0.003239017	0.036915802	7.00E-04	0.102475	induction of programmed cell death
GO:0035072	0.245653096	0.003239017	0.036915802	7.00E-04	0.102475	ecdysone-mediated induction of salivary gland cell autophagic cell death
GO:0035078	0.245653096	0.003239017	0.036915802	7.00E-04	0.102475	induction of programmed cell death by ecdysone
GO:0035081	0.245653096	0.003239017	0.036915802	7.00E-04	0.102475	induction of programmed cell death by hormones
GO:1904666	0.213128546	0.019690283	0.124482636	7.00E-04	0.102475	regulation of ubiquitin protein ligase activity
GO:0000271	0.17227768	0.038950734	0.195134143	7.00E-04	0.102475	polysaccharide biosynthetic process
GO:0060828	0.147328776	7.44E-05	0.001883572	5.00E-04	0.102475	regulation of canonical Wnt receptor signaling pathway
GO:0010623	0.103282871	0.004772342	0.047296908	6.00E-04	0.102475	developmental programmed cell death
GO:0030514	0.156580494	0.035952515	0.185085534	9.00E-04	0.119003226	negative regulation of BMP signaling pathway
GO:1901215	0.076751122	0.107128259	0.349679592	9.00E-04	0.119003226	negative regulation of neuron death
GO:0051048	0.113580766	0.071992997	0.279529736	0.001	0.12809375	negative regulation of secretion
GO:0005976	0.106242257	0.035103236	0.181624941	0.0012	0.149054545	polysaccharide metabolic process

Supplementary Table 6. List of Gene Ontology (GO) terms under convergent deceleration in social branches.

go_term_id	stat	*p*-value	*q*-value	permulation *p*-value	permulation *q*-value	go_term
GO:0009066	-0.184866908	0.002726685	0.033697557	0	0	aspartate family amino acid metabolic process
GO:0000096	-0.216099412	0.002795369	0.034237013	0	0	sulfur amino acid metabolic process
GO:0006555	-0.23675406	0.009577416	0.077491271	0	0	methionine metabolic process
GO:0001824	-0.267628644	0.003405195	0.037966076	0	0	blastocyst development
GO:0097502	-0.202736032	0.003845595	0.041520207	3.00E-04	0.068316667	mannosylation
GO:0009067	-0.217817114	0.012433539	0.09111905	7.00E-04	0.102475	aspartate family amino acid biosynthetic process
GO:1904375	-0.122012733	0.091440022	0.32095835	8.00E-04	0.113075862	regulation of protein localization to cell periphery

Supplementary Table 7. List of Gene Ontology (GO) terms for genes suggested by FADE with convergent amino acid substitutions.

go_term_id	p-value	q-value	go_term
GO:1904398	0.0000468	0.09127157	positive regulation of neuromuscular junction development
GO:0045887	0.0000468	0.09127157	positive regulation of synaptic assembly at neuromuscular junction
GO:1903532	0.0000162	0.09127157	positive regulation of secretion by cell
GO:0051047	0.0000199	0.09127157	positive regulation of secretion
GO:0042632	0.0000282	0.09127157	cholesterol homeostasis
GO:0055092	0.0000366	0.09127157	sterol homeostasis
