# Supplementary material for: Genomic signatures of recent convergent transitions to social life in spiders
Source: Nat Commun. 2022 Nov 22;13:6967. doi: 10.1038/s41467-022-34446-8 (PMC9681848; doi:10.1038/s41467-022-34446-8)
Supplement: Supplementary file 3 — Description of Additional Supplementary Files [file 41467_2022_34446_MOESM3_ESM.pdf]

Description of Additional Supplementary Files

File Name: Supplementary Data 1
Description: List of genes under convergent relaxation in social spiders

File Name: Supplementary Data 2
Description: List of genes under convergent intensification in social spiders.

File Name: Supplementary Data 3
Description: Gene ontology terms (Biological Processes, BP) under acceleration in social spiders.

File Name: Supplementary Data 4
Description: Gene ontology terms (Biological Processes, BP) under deceleration in social spiders.

File Name: Supplementary Data 5
Description: List of genes harbored convergent amino acid sites in social spiders.
