# Supplementary material for: Genomic signatures of recent convergent transitions to social life in spiders
Source: Nat Commun. 2022 Nov 22;13:6967. doi: 10.1038/s41467-022-34446-8 (PMC9681848; doi:10.1038/s41467-022-34446-8)
Supplement: Supplementary file 9 — Reporting Summary [file 41467_2022_34446_MOESM9_ESM.pdf]

nature portfolio

Corresponding author(s): Chao Tong, Timothy Linksvayer

Last updated by author(s): Oct 20, 2022

Reporting Summary

Nature Portfolio wishes to improve the reproducibility of the work that we publish. This form provides structure for consistency and transparency in reporting. For further information on Nature Portfolio policies, see our Editorial Policies and the Editorial Policy Checklist.

Statistics

For all statistical analyses, confirm that the following items are present in the figure legend, table legend, main text, or Methods section.

n/a	Confirmed	
☐	☒	The exact sample size (*n*) for each experimental group/condition, given as a discrete number and unit of measurement
☐	☒	A statement on whether measurements were taken from distinct samples or whether the same sample was measured repeatedly
☐	☒	The statistical test(s) used AND whether they are one- or two-sided<br>*Only common tests should be described solely by name; describe more complex techniques in the Methods section.*
☐	☒	A description of all covariates tested
☐	☒	A description of any assumptions or corrections, such as tests of normality and adjustment for multiple comparisons
☐	☒	A full description of the statistical parameters including central tendency (e.g. means) or other basic estimates (e.g. regression coefficient) AND variation (e.g. standard deviation) or associated estimates of uncertainty (e.g. confidence intervals)
☐	☒	For null hypothesis testing, the test statistic (e.g. *F*, *t*, *r*) with confidence intervals, effect sizes, degrees of freedom and *P* value noted<br>*Give P values as exact values whenever suitable.*
☐	☒	For Bayesian analysis, information on the choice of priors and Markov chain Monte Carlo settings
☐	☒	For hierarchical and complex designs, identification of the appropriate level for tests and full reporting of outcomes
☐	☒	Estimates of effect sizes (e.g. Cohen's *d*, Pearson's *r*), indicating how they were calculated

Our web collection on statistics for biologists contains articles on many of the points above.

Software and code

Policy information about availability of computer code

Data collection	No software was used for data collection.
Data analysis	Publicly available softwares, including FastQC v1, Trimmomatic v.0.39, Trinity v.2.6.5, CD-HIT v.4.8.1, BUSCO v.5.1.2, DIAMOND v.0.9.29, OMA, BLAST, OrthoDB, HaMStR v.1.0, eggNOG-mapper v2, trimAl v1.2, clustalo, ModelFinder, RAxML v8.2, rnaSPAdes, PEP_scaffolder, L_RNA_scaffolder, PAL2NAL v.14, PAML 4.7a, HyPhy 2.5, RELAX (HyPhy 2.5), GOATOOLS, RERconverge (R package), phangorn (R package)<br><br>All scripts required to perform all analyses are publicly available on Github at github.com/jiyideanjiao/Social_Spider_Evolutionary_Genomics

For manuscripts utilizing custom algorithms or software that are central to the research but not yet described in published literature, software must be made available to editors and reviewers. We strongly encourage code deposition in a community repository (e.g. GitHub). See the Nature Portfolio guidelines for submitting code & software for further information.

Data

Policy information about availability of data

All manuscripts must include a data availability statement. This statement should provide the following information, where applicable:

- Accession codes, unique identifiers, or web links for publicly available datasets
- A description of any restrictions on data availability
- For clinical datasets or third party data, please ensure that the statement adheres to our policy

Raw and processed transcriptome data have been deposited in NCBI under the project PRJNA685164. We also used available spider genome and transcriptome data which were downloaded from NCBI, including Acanthoscurria geniculata (GCA_000661875.l), Anelosimus studiosus (GCA_008297655.l), Stegodyphus mimosarum (GCA_000611955.2), Stegodyphus dumicola (GCA_010614865.l), Stegodyphus africanus (SRR7062696), Stegodyphus lineatus (SRR7062695), Theridion grallator (SRR960715, SRR960716, SRR960718, SRR960719, SRR960611,SRR960612,SRR960614,SRR960615,SRR960616), Heteropoda sp. ATS12 (SRR6425926). In addition, we used four calibration time from Timetree (www.timetree.org) and a recent published literature 32, including Stegodyphus mimosarum - Stegodyphus dumicola (8 to 15 Mya), Stegodyphus mimosarum - Stegodyphus lineatus (13 to 23 Mya), Anelosimus guacamayos - Anelosimus oritoyacu (1.4 to 2.7 Mya), Anelosimus eximius to Anelosimus domingo (3.7 to 6.3 Mya).

Human research participants

Policy information about studies involving human research participants and Sex and Gender in Research.

Reporting on sex and gender	*Use the terms sex (biological attribute) and gender (shaped by social and cultural circumstances) carefully in order to avoid confusing both terms. Indicate if findings apply to only one sex or gender; describe whether sex and gender were considered in study design whether sex and/or gender was determined based on self-reporting or assigned and methods used. Provide in the source data disaggregated sex and gender data where this information has been collected, and consent has been obtained for sharing of individual-level data; provide overall numbers in this Reporting Summary. Please state if this information has not been collected. Report sex- and gender-based analyses where performed, justify reasons for lack of sex- and gender-based analysis.*
Population characteristics	*Describe the covariate-relevant population characteristics of the human research participants (e.g. age, genotypic information, past and current diagnosis and treatment categories). If you filled out the behavioural & social sciences study design questions and have nothing to add here, write "See above."*
Recruitment	*Describe how participants were recruited. Outline any potential self-selection bias or other biases that may be present and how these are likely to impact results.*
Ethics oversight	*Identify the organization(s) that approved the study protocol.*

Note that full information on the approval of the study protocol must also be provided in the manuscript.

Field-specific reporting

Please select the one below that is the best fit for your research. If you are not sure, read the appropriate sections before making your selection.

☐ Life sciences ☐ Behavioural & social sciences ☒ Ecological, evolutionary & environmental sciences

For a reference copy of the document with all sections, see nature.com/documents/nr-reporting-summary-flat.pdf

Ecological, evolutionary & environmental sciences study design

All studies must disclose on these points even when the disclosure is negative.

Study description	We used a comparative genomic approach with 22 spider species representing seven independent and recent origins of sociality to answer a longstanding question: what genomic changes underpin the convergent evolution of sociality?
Research sample	Anelosimus eximius, Anelosimus domingo, Anelosimus oritoyacu, Anelosimus guacamayos, Anelosimus rupununi, Anelosimus elegans, Anelosimus arizona, Theridion nigroannulatum, Theridion differens, Theridion albidum, Theridion frondeum, Theridion murarium, Delena cancerides, lsopedella leai and Holconia hirsuta were collected from field in each sampling locations. Field-collected samples were morphologically inspected to confirm their identity. The samples were preserved in 95% ethanol prior to RNA extraction for transcriptome sequencing.<br>The available genomes or transcriptome of Acanthoscurria geniculata, Anelosimus studiosus, Stegodyphus mimosarum, Stegodyphus dumicola, Stegodyphus africanus, Stegodyphus lineatus, Theridion grallator, and Heteropoda sp. ATS12 were downloaded from NCBI (https://www.ncbi.nlm.nih.gov/).
Sampling strategy	The specimens represented each species were collected from each reported sampling locations. The number of individuals sampled for each spider species ranged from 3 -5.

Data collection	The spider specimens were collected from fields by LA, LSR and their lab members.
Timing and spatial scale	This study included the ethanol-preserved spider specimens that collected in 2009, 2014, 2015, 2017 and 2018 from Ecuador, USA and Australia.
Data exclusions	Individuals with poor RNA quality (RNA integrity number < 5) and poor assembly quality (BUSCO arachnid_odb9 < 70%) were excluded.
Reproducibility	No experiments were performed in this study.
Randomization	The individuals from a same colony represented each spider species were randomly collected for this study.
Blinding	This does not apply to our study.

Did the study involve field work? ☐ Yes ☒ No

Reporting for specific materials, systems and methods

We require information from authors about some types of materials, experimental systems and methods used in many studies. Here, indicate whether each material, system or method listed is relevant to your study. If you are not sure if a list item applies to your research, read the appropriate section before selecting a response.

Materials & experimental systems

n/a	Involved in the study
☒	☐ Antibodies
☒	☐ Eukaryotic cell lines
☒	☐ Palaeontology and archaeology
☐	☒ Animals and other organisms
☒	☐ Clinical data
☒	☐ Dual use research of concern

Methods

n/a	Involved in the study
☒	☐ ChIP-seq
☒	☐ Flow cytometry
☒	☐ MRI-based neuroimaging

Animals and other research organisms

Policy information about studies involving animals; ARRIVE guidelines recommended for reporting animal research, and Sex and Gender in Research

Laboratory animals	This study did not involve laboratory animals.
Wild animals	This study did not involve wild animals.
Reporting on sex	Female spiders
Field-collected samples	The field-collected samples were preserved in 95% ethanol prior to RNA extraction.
Ethics oversight	No ethical approval was required as studied invertebrates (arachnid).

Note that full information on the approval of the study protocol must also be provided in the manuscript.
